# Supplementary material for: Learning curve and surgical outcome of robotic assisted colorectal surgery with ERAS program
Source: Sci Rep. 2022 Nov 29;12:20566. doi: 10.1038/s41598-022-24665-w (PMC9709162; doi:10.1038/s41598-022-24665-w)
Supplement: Supplementary file 1 — Supplementary Information 1. [file 41598_2022_24665_MOESM1_ESM.zip › LSC Raw data-Ñ[▒K/062/H_ñw╝╨░O▒Kñσ.pdf]

正式摘要(電子簽章：0000001845545551)		
W55 / W55 ☐英文標題(注意:無英文姓名-請補病人基本檔!)	☐【讀取】憑證	

臺中榮民總醫院

出院病歷摘要

轉入號： 轉出號：

[redacted]

(8)流水編號	
(9)入院日期	109/01/14 (2020) 直腸外科 W55 - 265
(10)轉科日期	
(11)出院日期	109/01/23 (2020) 住院天數計 9 日

診斷

- 入院 :colon cancer, asending colon, cT3N0M0
- 出院 :colon cancer, asending colon, cT3N0M0, s/p laparoscopic right colectomy and anastomosis on 2020/1/15 pT3N1b (2/26)M0

主訴

- blood tinged stool for about 1 month

病史

- This 67-year-old man has history of
  1. Anterior lower gum ca, pT1NxM0, stage I-> s/p combine resection of oral cancer(Tumor wide excision + Rt FND, level I-IV) + marginal mandibulectomy + Rt forarm free flap reconstruction on 2014/3/4
  2. Tongue Ca s/p op at Sa-Ton hospital in 2009-03 /c left neck recurrence s/p L't neck dissection in 2009-12 s/p CCRT
  3. DM
  4. HTN
  5. Gouty arthritis
  6. colon polyps, s/p polypectomy for many times, pathology: many adenoma and 1 adenocarcinoma in 2014

  According to the patient, he suffered from blood tinged stool for about 1 month. He came to our CRS OPD. Colonoscopy showed multiple polyps over S-colon and D-colon, and the pathological results of polypectomy biopsy reveal adenocarcinoma 80cm from anal verge. Abdominal CT showed tumor about 2.5cm at ascending colon, and the whole body bone scan showed FDG uptake at hepatic flexure of the colon. There were no signs of liver or lung metastasis or lymph node invasion. Under the impression of colon cancer, asending colon, cT3N0M0, the patient was admitted for surgery.

體檢發現

- Evaluation

PE:

Tumor marker CEA CA-199
2019/12 5.2

image
2019/12 abd CT a colon
2020/1 PET A COLON CA.

colonsocopy
2019/12: Colonoscopy up to hepatic flexure, ulcerative tumor with lumen narrowing, scope can't pass. s/p biopsy.
Mutiple polyp over S-colon and D-colon. s/p biopsy.
Adenoma at 13cm FAV, s/p polypectomy.

SE
2018

Dx:

- General appearance :
  - easy looking , conscious clear , without cardiopulmonary distress
  - body weight : 80.9 kg , height : 160.7 cm
  - Vital signs自動帶入date 體溫(BT) 脈搏(P) 呼吸(R) 血壓(BP) 身高(BH) 體重(BW) BMI
    20200114 36.6℃ 71次/分 20次/分 129/75mmHg 161.4cm 80.5kg 30.9
  - Vital signs: B.P. 129/75 mmHg , P.R. 71 /min , R.R. 20 /min , B.T. 36.6 ℃
- HEENT :
  - Eyes: no anemic conjunctiva, no icteric sclera, normal EOM
  - ENT: no otorrhea, no rhinorrhea, no erythematous mucosa, no injected tonsil, no deformity
- Neck: supple, freely movable, trachea midline,no deformity, no engorged jugular vein , no palpable thyroid or lymph node enlargement
- Chest:
  - Inspection: Grossly normal, symmetric and fully expansion, no accessory muscle used, no operation scar, no spider angiomata, no gynecomastia
  - Palpation: no palpable mass or nodule, no subcutaneous emphysema, no tenderness
  - Ausculation: clear breathing sound
- Heart:
  - Palpation: no thrill, no heave
  - Percussion: normal heart size
  - Ausculation: regular heart beat, normal S1, S2, no gallop, no murmur

- Extremities: No deformity, full ranged of motion without limitation, no clubbing finger or nail cyanosis, no pitting edema, no ulcer or pigmentation, no obvious skin lesion, no varicose vein, no bone, joint or muscle tenderness

CRS CONDITION

- Karnofsky performance status: (100) Normal, no complaints, no evidence of disease
- PE of Abdomen:
- CEA: 5.35 ng/ml
- Imaging Studies:
- Colonoscope report: adenocarcinoma 80cm from anal verge
- Sono , CT of abdomen and chest Films:檢驗項目 :CT ABDOMEN-SURVEY 報告日期 :1081227

主 訴：
Result:
Finding:
Imaging Report Form for Colorectal Carcinoma (AJCC 8th)
1. Imaging Date: 20191224
Imaging Modality, Imaging by
CT scan
Imaging Protocol:
Post-contrast computed tomography scan of whole abdomen was performed with oral contrast ingestion
2. Tumor location / Size
Location:
Ascending
Size:
Measurable: 2.5 cm (largest diameter)
3. Tumor invasion
Tumor within colonic or rectal wall
4. Regional nodal metastasis
No
5. Distant metastasis (In this study)
No or Equivocal
6. Other findings:
> Ascending colon cancer for evaluation
> Focal irregular wall thickening over ascending colon(axial 32) is compatible with colon cancer.
> Bilateral renal cysts up to 1.8cm in size
> A fat contained lesion with sclerotic margin over left iliac bone, in favor of lipoma.
> No definite focal lesion in liver parenchyma, spleen, pancreas, gallbladder, bilateral adrenal glands
> No significant lymphadenopathy in the abdomen or retroperitoneum identified.
Impression:
Ascending colon cancer, cT1-2N0M0

- Figures:
  1. colon & rectum

手術日期及方法

- 手術日期及方法20200115 Laparoscopic right colectomy and anastomosis

住院治療經過

- After admission, preoperative survey showed no contraindication of surgery. laparoscopic right colectomy and side to side anastomosis was done on 2020/1/15. Emperic antibiotics with cefazolin, gentamycin and metronidazole were prescribed, and NPO with fluid supplement were kept. NG tube was removed on 1/16. Intermittent low grade fever were noted during 1/16-1/17 but subsided spontaneously. There were few whitish sputum, and the patient could cough it out. Since there was flatus, the patient started trying water on 1/18 then B-I diet on 1/20. There were no nausea or vomiting, and stool passage were noted on 1/21, then he started trying B-II diet. Stitches and penrose drain were removed on 1/22. Under relatively stable condition, the patient was discharged. We'll follow up the pathologic results.

合併症

- nil

一般檢查

- 累積報告：

DATE	ALB	TP	BIL.T	BII.D	ALKP	AST	ALT	LDH	CHOL	TG	HDL-C	LDL	UA	NH3	AMY
1081225	4.2		0.36	0.11	204	19	17	196							
1090114	4.7		0.5				12								
1090115	4.0		0.48	0.17	129	13	10								
1090117	3.6		0.6				5								
1090123	3.3		0.39				3								

DATE	WBC	RBC	HGB	HCT	MCV	PLT	BLS	PRO	MY	BAND	NEUT	LYM	MON	EOS	BAS	ANC
1081225	6720	4.18	11.0	34.4	82.3	328					58.5	32.9	7.3	1.0	0.3	3931
1090114	8410	4.34	10.7			333					62.9	27.0	8.3	1.4	0.4	5290
1090115	9770		9.6	30.5		263					81.5	13.8	4.4	0.1	0.2	7963
1090117	10950	4.07	10.1								78.8	14.0	6.7	0.3	0.2	8629
1090123	7120	4.30	10.2			338					70.2	18.8	7.7	2.7	0.6	4998

DATE	NA	K	CL	CA	BUN	CREAT	Mg	P	CRP	ESR	PCT	LIPASE	LACTATE
1081225	140	4.2		9.3	17	1.32							
1090114	138	4.6	101	9.7	23	1.52							
1090115	139	3.8		8.3	19	1.18							
1090117	135	4.0	101	8.2		0.96			10.029				
1090123	135	4.5	99	8.4	12	1.07							

特殊檢查

病理報告

- 活體組織切片病理報告　手術日期：**108/12/19**　病理號：**S0845498**

- 診斷醫師:孫志豪 報告日期:108/12/23

Pathologic diagnosis:
1. Colon, 80 cm from anal verge, labeled "tumor", "A", endoscopic biopsy --- Adenocarcinoma, moderately differentiated.
2. Colon, 30 cm from anal verge, "B", polypectomy and biopsy --- Tubular adenoma.
3. Colon, 20 cm from anal verge, "C", polypectomy and biopsy --- Tubular adenoma.
4. Colon, 15 cm from anal verge, "D", polypectomy and biopsy --- Tubular adenoma.
5. Mucosa, labeled "colon, 13 cm from anal verge", "E", polypectomy and biopsy --- Tubular adenoma.
Prognostic and predictive factor:
1. Degree of dysplasia: Low grade (for specimen B, C, D, and E).
2. Margin of stalk: Cannot be well evaluated (for specimen B, C, D, and E).
Gross description:
The specimen consists of 1) 3 pieces of tan soft tissue, up to 0.2x0.2x0.2 cm, labeled as "80 cm". 2) 2 pieces of tan soft tissue, up to 0.2x0.2x0.2 cm, labeled as "30 cm". 3) a piece of tan soft tissue, 0.3x0.2x0.2 cm, labeled as "20 cm". 4) a piece of tan soft tissue, 0.3x0.1x0.1 cm, labeled as "15 cm". 5) a piece of tan soft tissue, 0.5x0.3x0.2 cm, labeled as "13 cm". All for section: A) specimen A B) specimen B C) specimen C D) specimen D E) specimen E.
Microscopic description:
Section A shows colon mucosa with invasive moderately differentiated carcinoma exhibiting glandular differentiation. Sections B, C, D, and E show colon or colorectal mucosa with tubular glands proliferation with dysplasia.
Comment:
The diagnosis of adenocarcinoma has been concurred in intradepartment consensus meeting on 2019/12/23.
#T-59300_2 #M-81403_2 2 1340 00CM0F
#T-59300_2 #M-82110_2 0 1340 000000

活體組織切片病理報告　手術日期：**109/01/15**　病理號：**S0901893**

診斷醫師:楊啟順 報告日期:109/01/23
Pathologic diagnosis:
1. Colon, terminal ileum, and appendix, LAPA right hemicolectomy --- Adenocarcinoma, moderately differentiated, of ascending colon. Tumor invaded to mesocolic soft tissue and metastasized to mesocolic lymph nodes (2/26). The appendix, ileocecal valve, bilateral cut ends and radial margin are free of tumor.
2. Soft tissue, middle colic root, dissection --- Unremarkable lymph node (0/1).
Ancillary study for diagnosis:
IHC stains for MLH1, MSH2, PMS2 and MSH6 are done on section G. All above markers are preserved.
Prognostic and predictive factor:
1. Tumor size: 4x3.5 cm.
2. Depth of invasion: Mosocolic soft tissue.
3. Histologic grade: G2: Moderately differentiated.
4. Angiolymphatic invasion: Not identified.
5. Perineural invasion: Not identified.
6. Tumor deposits (discontinuous extramural extension): Not identified.
7. Surgical margins: a) Proximal Margin: Uninvolved by invasive carcinoma, distance of tumor from margin: 16 cm. b) Distal Margin: Uninvolved by invasive carcinoma, distance of tumor from margin : 4 cm. c) Radial or Mesenteric Margin: Uninvolved by invasive carcinoma, distance of tumor from margin: 3 cm.
8. Lymph node status: Involved (2/27).
9. Extranodal involvement: Not identified.
10. Tumor regression grading S/P CCRT: No known presurgical therapy.
11. Type of polyp in which invasive carcinoma arose: Not identified.
12. Additional pathologic findings: Not identified.
13. Pathological TNM Stage: pT3N1b (According to the eighth edition, 2017, American Joint Committee on Cancer Staging Guidelines for Tumors / 2017 CAP guideline).
14. TNM descriptors: Not applicable.
Gross description:
The specimen consists of 1) a segment of colon, 18 cm in length, and 7 cm in proximal circumference, 7 cm in distal cut end circumference, and a segment of terminal ileum, 6 cm in length, and 3 cm in cut end circumference. An unremarkable appendix, 6 cm, is noted. A gray tan firm annular elevated and centrally ulcerative tumor, 4x3.5 cm, is noted, 4 cm to the distal cut end. On cross section, the tumor invaded to the mesocolon with 3 cm to the radial margin grossly. The appendix, ileocecal valve, and both cut ends are free of tumor. 2) "middle colic root", a piece of yellow soft tissue, 4x3x2 cm. Representative part for section: A) specimen 2 B) proximal cut end C) distal cut end D) ileocecal valve E) appendix F) radial margin G-K) tumor L-N) lymph nodes.
Note:
1. The interpretation of MMR panel and recommended studies are as below: (1) No loss of nuclear expression of MMR proteins: low probability of microsatellite instability-high (MSI-H) . (2) Please note: There are exceptions to the above IHC interpretations. These results should not be considered in isolation, and clinical

- correlation with genetic counseling is recommended to assess the need for germline testing.
  2. This case has been peer reviewed by two doctors.
  #T-59420_2 #M-81403_2 2 1105 S0000F
  #T-C4520_2 #M-81406_2 9 1105 000000
  #T-59200_2 #M-09183_2 0 1105 000000

其它

出院時情況

- 3.改門診治療

出院指示

藥名	劑量	劑量單位	用法	頻次	數量	數量單位	現況	天數	單位
Ultracet tab	1	TAB	PO	BIDPRN	14	TAB	TH	7	天

出院計劃

主治醫師 2222H 林俊余 / 住院醫師 2350L 陳威遠

正式摘要(電子簽章：**000000184554551)**

W55 / W55

doctorRank:B
